# Biodegradable Metals for Cardiovascular Stent Application: Interests and New Opportunities

**DOI:** 10.3390/ijms12074250

**Published:** 2011-06-29

**Authors:** Maryam Moravej, Diego Mantovani

**Affiliations:** Laboratory for Biomaterials and Bioengineering, Department of Mining, Metallurgy and Materials Engineering & University Hospital Research Center, Laval University, Québec City, G1V 0A6, Que., Canada; E-Mail: maryam.moravej.1@ulaval.ca

**Keywords:** coronary stents, biodegradable stents, metallurgical processes, electroforming

## Abstract

During the last decade, biodegradable metallic stents have been developed and investigated as alternatives for the currently-used permanent cardiovascular stents. Degradable metallic materials could potentially replace corrosion-resistant metals currently used for stent application as it has been shown that the role of stenting is temporary and limited to a period of 6–12 months after implantation during which arterial remodeling and healing occur. Although corrosion is generally considered as a failure in metallurgy, the corrodibility of certain metals can be an advantage for their application as degradable implants. The candidate materials for such application should have mechanical properties ideally close to those of 316L stainless steel which is the gold standard material for stent application in order to provide mechanical support to diseased arteries. Non-toxicity of the metal itself and its degradation products is another requirement as the material is absorbed by blood and cells. Based on the mentioned requirements, iron-based and magnesium-based alloys have been the investigated candidates for biodegradable stents. This article reviews the recent developments in the design and evaluation of metallic materials for biodegradable stents. It also introduces the new metallurgical processes which could be applied for the production of metallic biodegradable stents and their effect on the properties of the produced metals.

## 1. Rational and History

Biomaterials used for implants can be metals, ceramics, polymers and composites. Metals have high impact strength, high wear resistance, high ductility and the capacity to absorb high strain energy (toughness) compared to other materials. These properties make metals suitable candidates for orthopedic load-bearing application and fixation devices such as joint replacement, bone plates and screws, as well as dental implants, pacer and suture wires, and coronary stents [1,2]. The early use of metals as biomaterials has been reported since late 18th century when Fe, Au, Ag and Pt were used as wires and pins to fix bone fractures [1]. The application of metallic biodegradable implants started shortly after the discovery of elemental magnesium by Sir Humphrey Davy in 1808. In 1878, the first implantation of Mg wires as ligatures to stop bleeding vessels of three human patients was performed by the physician Edward C. Huse. He observed that the corrosion of Mg was slower *in vivo* and that the time period until complete degradation was dependent on the size of the Mg wire used [3]. It is traditionally believed that metallic implants should be made from corrosion-resistant metals such as medical grade stainless steel and Ti alloys. However, the principal focus in modern implant development is on developing devices that are strong, durable and at the same time more acceptable by the body. With the development of biodegradable implants, the concept of biomaterials has shifted from purely mechanical replacement devices towards true biological solutions [4]. Although corrosion is generally considered as a failure in metallurgy, the corrodibility of certain metals including magnesium and iron can be an advantage in their application as biodegradable implants. These materials do their job while healing and new tissue forming occur and degrade thereafter. These metals have revolutionized the orthopedic and cardiovascular surgery by combining engineering and medical requirements for implants. Moreover, the application of biodegradable metals in temporary implantable nanomedical devices such as sensors and actuators has been recently proposed [5].

## 2. Coronary Stents

A stent is a small mesh-like tubular scaffold which is placed and then expanded inside the coronary artery to keep the lumen open. At present, stenting is performed during about 60 percent of balloon angioplasty cases. In this process, the stent is mounted on the balloon and is expanded and positioned by inflating the balloon. The expansion of the stent pushes it up against the artery wall and when the balloon is deflated the stent remains in place and holds the artery open [6]. Stenting can considerably reduce the risk of restenosis after the angioplasty, however, in about 25% of stenting cases, the problem of restenosis can still remain, which is called in-stent restenosis (ISR) [7].

## 3. Biodegradable Stents

The current stent technology is based on the use of permanent stent made from corrosion-resistant metals. The implantation of bare metal stents, mainly fabricated from 316L stainless steel (316L SS), Nitinol and cobalt-chromium alloy, has shown tremendous superior effects in various kinds of clinical situations, especially in the field of percutaneous coronary intervention, compared to simple balloon angioplasty. Stent implantation can reduce the subsequent restenosis after angioplasty by utilizing its scaffolding effect and preventing the lumen shrinkage as a result of pathologic remodeling [8]. However, permanent metallic implants have specific drawbacks which limit their more widespread use. These limitations include long-term endothelial dysfunction, delayed re-endothelialization, thrombogenicity, permanent physical irritation, chronic inflammatory local reactions, mismatches in mechanical behavior between stented and non-stented vessel areas, inability to adapt to growth in young patients, and importantly non-permissive or disadvantageous characteristics for later surgical revascularization [9].

Since the major effect of stent implantation is provided by its scaffolding effect, it is required to last for 6–12 months during which arterial remodeling and healing is achieved. After this period, the presence of stent within the body cannot provide any beneficial effects. Thus, the development of biodegradable stents, which can fulfill the mission and step away, is the logical approach [8–11]. The material for biodegradable stents is requested to have at least the following characteristics: it must be biocompatible; degradation products of the material must also be biocompatible, the material must stay in the place for several months before its complete bioabsorption and the radial force of the resultant stent must be enough for scaffolding effect during the requested period [8]. Based on these requirements, two metallic elements including iron and magnesium have been investigated for this application.

### 3.1. Iron-Based Biodegradable Stents

**Figure 1:**
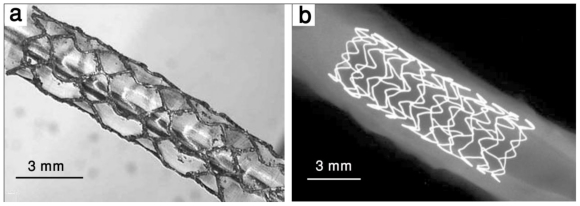
Biodegradable iron stents: (**a**) NOR-I stent expanded to 3.5 mm diameter and (**b**) X-ray photograph of iron stent (Biotronik, Erlangen, Germany ) after implantation in porcine coronary artery, adapted from References [16] and [15], respectively.

The first biodegradable metallic stent was fabricated from Armco^®^ iron (Fe > 99.8%) and implanted in descending aorta of New Zealand white rabbits in 2001 [10]. Iron can interconvert between ferric (Fe^2+^) and ferrous (Fe^3+^) ions by accepting and donating electrons quite readily, which makes it a useful component for cytochromes, oxygen-binding molecules (hemoglobin and myoglobin), and many enzymes. Even though iron is essential to life, its excess or deficiency can be deleterious [12]. The results from the implantation of the first iron stent showed no significant evidence of either an inflammatory response or neointimal proliferation, and organ examination did not reveal any systemic toxicity. There was also a maintained stent patency and no adverse events during a 6–18 month follow-up period [10]. Iron is also an interesting candidate for biodegradable stent in terms of its mechanical properties. It has a high radial strength because of its higher elastic modulus. This can be helpful in making stents with thinner struts. Iron has also high ductility which can be helpful during the implantation of stent when the stent is plastically deformed [13]. The results from the implantation of iron stent showed that the stents maintained their mechanical properties during the implantation without any failure [10]. In 2006, another study was performed to evaluate the safety of corrodible iron stent in a peripheral stent design (6–12 mm diameter) in a slotted tube design [14]. Iron stents were implanted into the descending aorta of minipigs which were followed for 1–360 days and 316L SS stents were implanted as reference. The results showed no difference with regard to the amount of neointimal proliferation between 316L SS and iron stents. Also no signs of iron overload or iron-related organ toxicity were observed. Adjacent to the iron stent struts, there was no evidence for local toxicity due to corrosion products. It was concluded that iron is a suitable metal for the production of a large-size degradable stent. However, the implantation of Armco^®^ iron stent showed that the stents did not corrode completely during the follow up period and therefore, faster degradation rate is desirable for iron and further studies have to focus on the modification of the composition and design of the stent to expedite the degradation process [10,14]. In a more recent study, iron stents were deployed in the coronary arteries of juvenile domestic pigs [15]. Cobalt chromium stents were also implanted for comparison. Short-term effects of the implanted stent were investigated after 28 days. Results showed that iron stents started to show signs of degradation without evidence of stent particle embolization or thrombosis without traces of excess inflammation, or fibrin deposition. At 28 days, the surface of the iron stent struts was black to brown and the vascular wall adjacent to the iron stent had a brownish tinge. There were no statistically significant differences in any of the measured parameters between segments implanted with iron and cobalt chromium stents [15]. Because the study was limited to a short period after implantation, no conclusion could be drawn about the degradation rate of iron stent. However, the results of long-term implantation of iron stent showed that future efforts have to focus on the acceleration of the degradation rate. The suggested mechanisms were either using iron-based alloys with a more pronounced corrosion rate or increasing the surface of the stent along with reduction of the strut thickness and modification of the stent design [10,14]. Figure 1 shows two iron biodegradable stents implanted in animal models.

Based on these findings, several investigations have been performed to increase the degradation rate of iron-based materials while maintaining its interesting mechanical properties. These studies focused on the development of new alloys or microstructures from a material science point of view to achieve desired stent properties. Table 1 presents the data on microstructure (grain size), mechanical properties and *in vitro* degradation rate of different iron-based materials for biodegradable stents compared to those of Armco^®^ iron which had been investigated *in vivo*. The properties of 316L SS are also presented as reference since it is considered as the gold standard metal for clinical stent implantation.

Hermawan *et al.* [19–21,28] were the first to investigate the effect of alloying elements on the properties of iron for biodegradable stents. Mn was selected as the alloying element with four different contents from 20 to 35 wt%. The rational was to produce an antiferromagnetic Fe-Mn alloy with magnetic resonance imaging (MRI) compatibility, mechanical properties comparable to those of 316L SS, and higher degradation rate than that of pure iron. Fe-Mn alloys were produced by powder metallurgy and several rolling-sintering steps were carried out to achieve the desired microstructure and properties. The results showed that Fe-30Mn and Fe-35Mn alloys contained single austenitic phase with mechanical properties comparable to those of 316L SS and the degradation rate more than two times higher than that of pure iron. These alloys showed a low inhibition to metabolic activity of fibroblast cells in cell viability studies. Based on these results, it was suggested that the alloys are recommended for further *in vitro* biodegradation and *in vivo* implantation studies [19–21,28]. Shcinhammer *et al.* [22] also reported a design strategy for the development of new biodegradable Fe-based alloys with enhanced degradation rate compared to pure iron, and suitable strength and ductility. The influence of alloying elements including Mn and Pd on the electrochemical modification of the Fe and the controlled formation of noble intermetallic phases was deployed. Such intermetallic phases were responsible for both an increased degradation rate and enhanced strength. Mn lowered the standard electrode potential of the alloy, while Pd formed noble (Fe, Mn)Pd intermetallics that acted as cathodic sites thereby increasing the degradation rate. The developed Fe-10Mn-1Pd alloys revealed a degradation resistance one order of magnitude lower than observed for pure iron. Moreover, the mechanical performance of the alloy was shown to be adjustable not only by the choice of alloying elements but also by heat treatment procedures. Thus, this new alloy offers an attractive combination of electrochemical and mechanical characteristics considered suitable for biodegradable stent applications. The investigation of the effect of alloying elements on biodegradability and biocompatibility of iron for biodegradable stents was also carried out by Liu *et al.* [25]. They produced six Fe-X binary alloys with the concentration of X being 3 at.% to investigate the effect of Mn, Co, Al, W, Sn and B elements. High carbon steel 1070 (3 at.% C content) and free cutting steel 1119 (0.5 at.% S content) were also chosen to investigate the effects of the alloying elements C and S on iron. The alloy ingots were produced by casting in vacuum induction furnace and the alloy properties were studies in both as-cast and as-rolled states. Tensile test, corrosion testing (including potentiodynamic polarization, static immersion and dynamic degradation), cytotoxicity testing and hemocompatibility testing were performed on the iron alloys. The alloying elements Mn, Co, W, B, C, and S were found to improve the yield and ultimate strength of iron in the as-rolled group and increase the difference between the yield strength and ultimate strength of iron, whereas the alloying element Sn led to a severe reduction in the mechanical properties. According to the results of corrosion testing, localized corrosion was the main mode of corrosion of pure iron and the Fe-X binary alloys. The corrosion rates of pure iron and the Fe-X binary alloys were of the same order of magnitude. The pure iron and Fe-X binary alloy extracts decreased the viabilities of L929 murine fibrosarcoma cells and vascular smooth muscle cells (VSMC) compared to 316L SS but showed no significant cytotoxicity to ECV304 endothelial cells, except for the Fe-Mn alloy. Although there was a slight increase in percentage hemolysis for pure iron and the Fe-X binary alloys compared with 316L SS, all hemolysis percentage was less than 5%. It was concluded that, the elements Co, W, C, and S are suitable as alloying elements for iron biomaterials on a comprehensive consideration of the improved mechanical properties, appropriate corrosion rates and good biocompatibility. In a more recent work, Liu *et al.* [26] developed a new shape memory iron-based alloy, Fe30Mn6Si, as a potential degradable biomaterial for stents and compared its microstructure, mechanical properties, degradation, citotoxicity and hemolysis to those of pure iron and Fe-30Mn alloy. Fe30Mn6Si alloy was produced by casting in a vacuum induction furnace and subsequently solution treated at 850 °C for 1 h and water quenched to enhance its shape memory effect. It was found that Fe30Mn6Si alloy consisted of ɛ-martensite and γ-austenite phases at room temperature and had higher mechanical properties than that of the pure iron and Fe30Mn alloy. The corrosion rate of Fe30Mn6Si alloy was higher than that of Fe30Mn alloy but lower than pure iron. Fe30Mn6Si alloy showed to inhibit the metabolic activity of human endothelial cells (ECV304) and rodent VSMC to a higher extend compared to pure iron. However, it was noticed that the ECV304 cell viability began to rise from day 2 to day 4, which is a good sign for cellular application. Moreover, a little increase in hemolysis value of Fe30Mn6Si alloy sample was observed in comparison with 316L SS but the hemolysis percentage was less than 2% and the alloy was considered to be non-hemolytic according to ASTM-F756-08 [29].

Another approach investigated to increase the degradation rate of pure iron was microstructural modification. The present authors developed an electroforming process for production of pure iron for biodegradable stents [23]. Mechanical properties, degradation behavior and cell viability of electroformed iron was investigated to evaluate the potential application of the material for cardiovascular stents [23,24]. Electroformed iron had a much finer microstructure compared to those of Armco^®^ iron (6 μm *versus* 40 μm) which resulted in superior yield and tensile strength. The elongation of as-electroformed iron was 8% which is a characteristic of electrodeposited materials. The elongation was increased to 18% after annealing at 550 °C due to stress relief and recrystallization. Electroformed iron also exhibited a highly oriented microstructure (texture) with the formation of columnar grains. The texture and grain size has been shown to influence the degradation behavior of the material [30]. Generally, the corrosion of electroformed iron was higher than that of Armco^®^ iron because of its smaller grains providing higher grain boundary area being more susceptible to the corrosive attack. The high density of microstructural defects in electroformed iron was also another reason for the higher corrosion rate. The corrosion mechanism of electroformed iron appeared to be uniform as no localized attack was observed after the degradation testing. Electroformed iron also showed to have no inhibition to cell metabolic activity of primary rat SMCs compared to 316L SS and Armco^®^ iron. The proliferation of SMCs however, decreased in contact with electroformed iron which could be potentially interesting for its application as stent to decrease in stent restenosis. Nie *et al*. [27] also investigated the effect of microstructural modifications on the properties of pure iron for stent application. The fabricated nanocrystalline pure iron rods by the equal channel angular pressure (ECAP) technique up to eight passes. The microstructure and grain size distribution, static immersion and electrochemical corrosion in simulated body fluid, cellular responses and hemocompatibility were investigated. The results indicated that nanocrystalline pure iron after severe plastic deformation would exhibit much stronger corrosion resistance than that of the microcrystalline pure iron. In cell cellular response studies, the interaction of different cell lines revealed that the nanocrystalline pure iron stimulated the proliferation of fibroblast cells more effectively and enhanced the preferable promotion of endothelialization while inhibiting the viability of VSMSc. In hemocompatibility studies, the burst of red cells and adhesion of the platelets were also substantially suppressed on contact with the nanocrystalline pure iron in blood circulation. Nie *et al*. [27] observed a clear size-dependent behavior of the cells associated with the grain size nature of the material and concluded that, in spite of its low degradation rate, nanocrystalline iron exhibits a good biocompatibility in terms of cell response and hemocompatibility and could be an interesting novel candidate for implants.

### 3.2. Magnesium-Based Biodegradable Stents

Magnesium is another attractive metal for biodegradable implants because of its low thrombogenicity and well-known biocompatibility. It is an essential trace element and has a high systemic toxic level which is about 7 to 10 millimols per liter of serum [16]. The use of magnesium as a biodegradable stent material was also based on the fact that it is a structural constituent of the tissue and essential element in the living organism. Magnesium is a substantial intercellular cation which is involved in more than 300 biological reactions of cell. Magnesium is also regarded as a non-carcinogenic element [31]. However, magnesium has a rapid degradation in aggressive chloride environments like body fluid. Rapid degradation of magnesium implant results in tissue overload with degradation products and this can lead to neointimal formation. Accelerated degradation of magnesium can also cause the loss of mechanical integrity in a short period which can limit its application as an implant material. Therefore, magnesium is alloyed with other elements such as aluminum, manganese and rare earth elements in order to decrease the degradation rate [32]. The first application of magnesium in cardiovascular applications dates back to the year 1878 when Huse used a Mg wire ligature successfully to stop bleeding vessels three times: once in a radial artery and twice in the operation for varicocele. Later in the 20th century, magnesium was used in several investigations as biodegradable material for connectors for vessel anastomosis and wires for aneurysm treatment [3]. However, Heublein *et al.* [33,34] were the first to investigate the idea of using magnesium alloys for cardiovascular stents. They selected AE21 alloy which has lower degradation rate compared to other magnesium alloys for an initial coronary animal study. The alloy was expected to have up to 50% mass loss during the first half-year of the implantation. The experiments were performed by implantation of stents into the coronary artery of eleven domestic pigs and the follow up procedure was performed at 10, 35 and 56 days after implantation. The histological analysis showed that AE21 magnesium stent induced a neointimal response, but this disadvantage was offset by later positive remodeling. There was not also any platelet deposition or thrombus at the endothelial sites after any of assessment intervals. Furthermore, a negligible inflammatory response was observed on evaluating each strut. The problem of AE21 stent was that its degradation occurred faster that the expected rate as the loss of mechanical integrity occurred between 35 and 56 days after implantation. Therefore, further improvements are necessary with respect to prolongation of the degradation and mechanical stability over a defined time. In addition, the short and long term local biocompatibility and bioreactivity of such alloys and their components before and during degradation need to be assessed. These investigations and experiments later resulted in the invention of a new generation of biodegradable stents in Biotronic Company [34].

**Figure 2:**
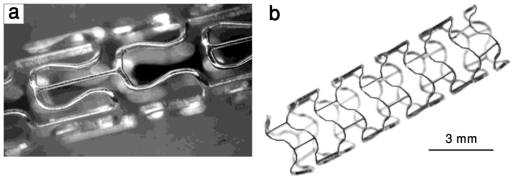
Lekton Magic coronary stent: (**a**) non-expanded and (**b**) expanded, adapted from Reference [16].

Di Mario *et al.* [35] reported the results of experimental implantation of Lekton Magic coronary stent (Biotronik, Bulach, Switzerland) made from WE43 magnesium alloy in the coronary artery of 33 mini-pigs. Figure 2 shows two images of LeKton Magic stent in non-expanded and expanded states. Peeters *et al*. [36] reported the results of the first clinical study of Lekton Magic coronary stent implantation in human for treatment of critical lower limb ischemia of 20 patients. The preliminary results showed that there were no symptoms of allergic or toxic reactions to the stent material. However, a more recent study on the implantation of the same biodegradable magnesium stent in the lower limb artery of 60 patients showed that although the biodegradable metallic stent technology can be safely applied, it did not demonstrate efficacy in long-term (6 months) patency over standard angioplasty in the lower limb vessels [37].

The First successful implantation of a biodegradable metal stent in human was performed by Zartner *et al.* in the left pulmonary artery of a preterm baby with a congenital heart disease [38]. The biodegradable stent selected for implantation was a magnesium stent 3 mm in diameter and 10 mm in length fabricated by Biotronik Company. The follow-up procedure showed the complete degradation occurred during 5 months and no in-stent obstruction or neointimal hypertrophy could be observed. This first result with a biodegradable magnesium stent implantation rescued a child from an extremely severe clinical problem and this case implicate that such stent technology may be more widely applicable in babies and children with different stent diameters and lengths.

In 2006, Waksman *et al*. [39] investigated the safety and efficacy of bioabsorbable WE43 magnesium alloy stents in porcine coronary arteries for a period of 3 months. There was no evidence of stent particle embolization, thrombosis, excess inflammation, or fibrin deposition and neointimal area was significantly less in magnesium alloy stent segments as compared with the stainless steel stent segments. They concluded that magnesium alloy stents are safe and are associated with less neointima formation; however, reduced neointima did not result in larger lumen. Later in 2007, the first results on clinical implantation of 71 WE43 magnesium stents in the coronary arteries of 63 patients were reported [40]. Follow-up included coronary angiography and intravascular ultrasound at 4 months and clinical assessment at 6 months and 12 months Angiography at 4 months showed an increased diameter stenosis of 17.0%. After serial intravascular ultrasound examinations, only small remnants of the original struts were visible, well embedded into the intima. Neointimal growth and negative remodelling were the main operating mechanisms of restenosis. This study showed that biodegradable magnesium stents can achieve an immediate angiographic result similar to the result of other metal stents and can be safely degraded after 4 months. However, modifications of stent characteristics with prolonged degradation and drug elution are still required and currently in development.

Besides *in vivo* implantation of the currently available magnesium alloy, investigations into the development and validation of new magnesium alloys have been performed in order to achieve higher mechanical properties and corrosion resistance. Generally, magnesium alloys have lower mechanical properties and faster degradation than iron based alloys. Information on the mechanical properties and degradation rate of different magnesium alloys investigated for biodegradable stents is presented in Table 2. The properties of 316 SS are presented as reference for comparison.

To control the biodegradation rate of Mg alloys stents, the surface of the stent is coated by biodegradable polymeric layers including PLGA and PLLA either in blank form of mixed with antiproliferative and antithrombosis drugs. These drugs can be released from the stent coating diffusion mechanisms or during polymer breakdown increasing the total degradation time of the stent [46–50]. AMS-3 stent from Biotronik Company is an Mg alloy stent coated with a fast-degradable polymer carrier with an anti-proliferative drug. This stent associates with developmental complexity due to the interactions of the degradation kinetics of the metallic platform, polymer carrier and the drug. The first animal trial in porcine model showed promising results in terms of safety and efficacy compared to bare AMS Mg stent [50]. However, more information is required on the *in vivo* interaction of the AMS-3 stent. Lu *et al*. [46] reported the fabrication biodegradable AZ81 Mg alloy stent coated with a composite multi-layer film for control of the biodegradation rate and drug release rate of the magnesium alloy. They also performed *in vitro* evaluation of the coated alloy and compared the results with those 316L SS. The first layer was composed of a micro-arc oxidation/poly-l-lactic acid (MAO/PLLA) composite coating which was shown to increase the corrosion resistance and control the biodegradation rate. PLLA effectively sealed the micro-cracks and micro-holes on the surface of the MAO coating to give controllable biodegradation of the AZ81 alloy. The drug release coating was composed of one Poly (d,l-lactide-co-glycolide)/paclitaxel (PLGA/PTX) layer and one pure PLGA blank layer without paclitaxel, and this coating also functioned to provide controlled biodegradation rate of the stent. The platelet adhesion test of the samples showed less platelet adhesion, aggregation, and activation on anticorrosion MAO/PLLA coating and the drug-eluting PLGA50/50 (8% PTX) coating than 316L SS. So, the modified samples had good blood compatibility. Although these results seem to be promising, further *in vivo* assessment of the coated Mg alloy is mandatory. Kondyurin *et al*. [51] has recently reported the in-water degradation behavior of a drug eluting PLGA layer coated on a 316L SS stent by ion implantation. It was found that PLGA films undergo crosslinking, carbonization and etching. The PLGA coating degrades in water by a hydrolysis reaction. The dewetting of PLGA coating under water was observed on metal surface. As a result, the coating was ruptured up to separate drops of PLGA on the surface. Therefore, careful assessment of the degradation behavior of this material should be carried out to prevent the release of separate particles in blood in case of stent implantation. It was reported that the dewetting process can be eliminated by an ion beam implantation treatment, when the ions penetrate through the whole PLGA coating.

From a materials science point of view, the mechanical properties and corrosion resistance of Mg alloys can be improved by the addition alloying elements especially rare earth elements and other metallic elements including lithium, zirconium and calcium. Lithium enhances ductility and formability of magnesium alloys by changing the lattice structure from hexagonal close packed (hcp) to body-centered cubic (bcc). Zirconium is an effective grain-refining agent in Al-free magnesium alloys which contributes to grain boundary strengthening. Calcium improves the alloy’s strength and creep resistance and acts as grain-refining agent. Moreover, it decreases the corrosion rate in trace amounts and is well tolerated in the human body since it is an essential cation [3,41,52]. Generally, Mg and its alloys have low density and stiffness close to those of cortical bone (*ρ* = 1.99 g cm^−3^ and *E* = 11.7–18.2 GPa). Its low Young’s modulus will be of benefit in reducing the stress shielding at the bone-implant interface, which makes Mg alloys more interesting candidates for biodegradable bone implants than in biodegradable stent applications [12]. Recently, newly-developed bio-absorbable magnesium alloys ZW21 and WZ21 containing Zn, Y, Ca and Mn as alloying elements showed fine and even microstructures with grains smaller than 10 μm, which generated exceptional plasticity of 17% and 20% at ambient temperature which make them interesting candidates for stent application [43–45]. These alloys also exhibited fairly homogeneous degradation behavior in physiologically simulated solutions and a higher resistance to corrosion than that of other magnesium alloys. *In vitro* cell tests using human umbilical vein endothelial cells indicated good cytocompatibility on the basis of the alloys’ extracts. Animal implantation studies of WZ21 alloy disc specimens was carried out by implantation in different abdominal tissues of pigs for 21 and 97 days. Although these preliminary results revealed a promising *in vivo* performance for the alloy, further studies should be conducted by the implantation of the alloy as vascular stent [43–45].

## 4. Fabrication Process for Biodegradable Stents

**Figure 3:**
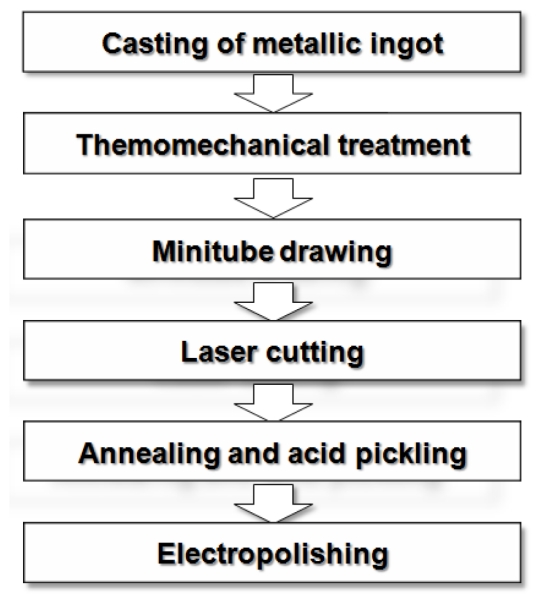
Fabrication process of cardiovascular stents.

The general fabrication process of cardiovascular stents including biodegradable metallic stents is illustrated in Figure 3. This process starts with the production of metallic ingots from which the stent will be made. Currently, most of surgical implant metallic materials including stents are fabricated by casting and thermomechanical treatment methods [53]. Metallic parts can be cast either directly into the shape of the component or as ingots that can be subsequently shaped into a desired form using other forming processes. From an economic standpoint, it would be desirable to form most metal components directly from casting, since subsequent operations such as forming; extruding, annealing and joining add additional expense. However, since the growth rate of crystalline phases is much higher in the liquid state than in the solid state, the microstructure of as-cast materials is much coarser than that of heat-treated or annealed metals. Casting is generally only used as the primary fabrication process and consequently, other forming and thermo-mechanical processes are performed to achieve the desired shape and mechanical properties [54]. Melting of most implant metals is performed under vacuum condition. The use of vacuum in melting operations is used not only to prevent reactions such as oxidation, but to prevent, and even remove, dissolved gases in the metals in order to avoid porosity [55]. In the case of stent materials, the selection of a melt source is extremely important. The nature and purity of the elemental material components mixed prior to melting, together with melt practice itself, have an influence on homogeneity, porosity and microcleanliness of the cast alloy [56]. The mechanical working of implant metals can be accomplished by various processes, including forging, rolling, and extrusion which are usually performed at elevated temperatures. Such hot working processes break down the cast structure and improve mechanical properties by plastic deformation and work hardening mechanisms. Optimal hot working temperatures are selected in the range that the alloy is easily workable and the surface oxidation in air is not too severe. Following hot working, metals are cold worked and heat-treated to obtain final dimensions with desired physical and mechanical properties [55,57]. The majority of biodegradable stents metallic materials reported in the literature have been produced by casting and thermomechanical treatment [10,15,22,25–27,35,38,40–42,52].

The next step is the fabrication of minitubes from which the stents are cut. Tubing is typically produced in either welded-redrawn or seamless form. Despite the robustness of modern welding equipment and continuous on line inspection systems, it is not possible to guarantee a fully defect-free weld without localized micro-contamination and segregation. Therefore, the tubing utilized for stents and other implantable devices should be seamless. Next, an adequate degree of control in the tube drawing process is required to ensure repeatable properties for the tubing. For example, while stainless steel exhibits a moderate sensitivity to processing parameters, Nitinol is more difficult to control and can show wide ranges in its properties, depending on processing [56]. Generally, stents are laser cut from metal minitubes. Laser cutting allows precise cutting of stent designs from tubes and because of its computer controlled mechanism, it is flexible in stent designs. After laser cutting, annealing is performed in order to relieve the residual stress produced during tube drawing and laser cutting and to improve the mechanical properties of the stent [58]. Annealed cut tubes are then acid pickled to remove the undesired part of cut tubes as well as burrs and debris. The final step in stent fabrication is electrolytic polishing or electropolishing which is the electrolytic removal of metal in a highly ionic solution. For biodegradable stents, it is performed to remove burrs and mechanical defects resulting from the heat ablation of the laser cutting, etching and forming steps and to achieve a smoother metal surface than chemical or acid etching alone [59].

### 4.1. New Fabrication Processes

As it was mentioned in the previous section, the majority of coronary stents are produced from metallic ingots fabricated by casting and thermomechanical treatment. Recently, the application of new processes has been investigated for biodegradable metallic stents. A review of these investigations is presented in this section.

#### 4.1.1. Powder Metallurgy

**Figure 4:**
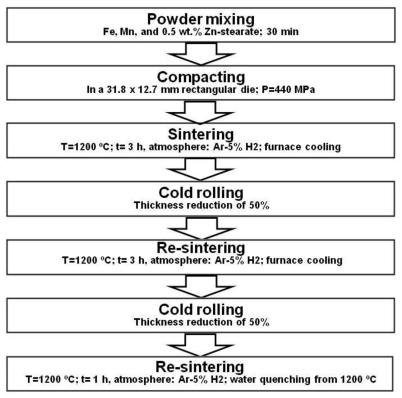
Fabrication process of Fe-35Mn alloy by powder metallurgy for biodegradable stents, adapted from Reference [20].

Powder metallurgy (PM) process has already been used for the fabrication of Nitinol permanent implant materials in the experimental basis. PM fabrication technique involves the compaction of powdered metal, followed by a heat treatment to produce a denser piece. Resulting density varies from process to process and the highest density (95%) has been achieved by Hot Isostatic Pressing (HIP). Elemental powders of Ni and Ti can be sintered using either combustion or thermal explosion process. Otherwise, Nitinol materials sintered from elemental powders are highly porous and may contain other intermetallic phases of Ti_2_Ni and TiNi_3_. The limitation of Nitinol PM processes appears to be the oxygen content which has been reported to exceed 3000 ppm. Oxygen at this level may negatively impact ductility and fatigue resistance [57]. Hermawan *et al.* [20] were the first to investigate the application PM to produce alloys for biodegradable stents. The production process of Fe-Mn alloys by PM is presented in Figure 4. The challenge in powder metallurgy, especially in sintering is to obtain a high density product and keep the grain size in the desired range. Higher sintering temperatures lead to higher densities but also result in grain growth. Therefore, the sintering parameters should be optimized. Also in this process the size, morphology and purity of metal powders, mixing time and conditions, pressing load and sintering atmosphere influence the properties of the products [60]. Fe-35Mn alloy produced by PM was single austenitic (γ) phase with interesting mechanical properties including high yield strength (234 MPa) and ductility (31%). Although porosity was observed in the microstructure of the alloy after the first sintering step, cold rolling and resintering cycles were found to significantly improve the densification and leave about 0.3% porosity and 2% MnO particles in the microstructure. The remaining porosity and MnO inclusions were shown to be advantageous for biodegradable Fe-35Mn alloy as it accelerates the degradation of the alloy compared pure iron [19–21].

**Figure 5:**
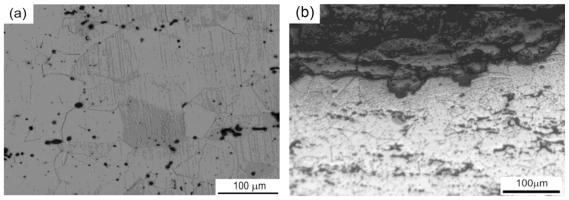
(**a**) Microstructure of PM Fe-35Mn alloy after PM processing and (**b**) Cross-section of the corroded alloy after dynamic degradation testing, adapted from Reference [21] and [28], respectively.

To highlight the effect of porosity resulted from PM processing on the degradation rate, Fe-35Mn alloy produced by PM could be compared to Fe-30Mn alloy produced by casting and thermomechanical treatment by Liu *et al*. [26]. The alloy produced by casting had higher ductility and was denser than that produced by PM. The result on potentiodynamic polarization corrosion testing showed a degradation rate of 0.12 mm y^−1^ of Fe-30Mn produced by casting while the corrosion rate of Fe-35Mn PM alloy was 0.44 mm y^−1^. The distribution of porosity in the microstructure of Fe-35Mn PM alloy and the resulting degradation form are presented in Figure 5.

As illustrated in Figure 5a, the distribution of pores in the microstructure resulting from PM could be observed. The corrosion mechanism of Fe-35Mn alloy after 3 months of dynamic degradation testing is presented in Figure 5b. It has been suggested that for PM Fe-35Mn alloy, the aligned porosity could stimulate a layer by layer degradation process, similar to the case of exfoliation corrosion, especially in the flowing solution of blood vessel where stents are implanted [28]. Therefore, PM can be an interesting processing method for production of iron-based biodegradable stents in order to obtain a porous microstructure and thereby increase the degradation rate. However, the control of the distribution of porosity should be acquired as the mechanical properties of the material are also influenced by the porosity.

#### 4.1.2. Electroforming

**Figure 6:**
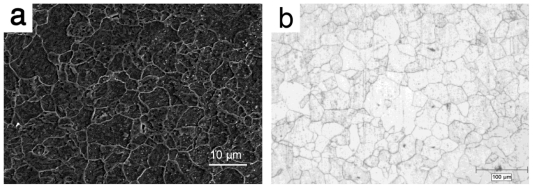
Microstructure of (**a**) annealed electroformed iron and (**b**) Armco^®^ iron.

The application of a novel technique, electroforming, generally used in microfabrication has been reported by the present authors [23]. Electroforming is the production of a thin metallic layer on a surface of a conductive substrate by electrolysis. The principle of electroforming is the same as that of electrodeposition. While electrodeposition is performed to produce a coating on a metallic surface to improve its properties, in electroforming, the metallic layer is separated from its substrate and used as a separate entity [61]. Electrodeposition have been previously used to apply coatings on the surface of metallic stents to alter their surface morphology, release drugs, enhance radio-opacity, and prevent corrosion [62]. However, it has never been explored for the fabrication of a structure for load-bearing applications such as biodegradable stents. Using electroforming to fabricate stent materials appears to be logical because this method which produces a metallic part by building up the structure of the material layer by layer could be an excellent method for manufacturing thin-walled products. This method is also well-known for production of nanocrystalline metals with grain sizes in the order of few nanometers [63]. In conventional metal working processes the metal is normally cast as a massive billet or ingot, which needs to be progressively reduced in size to yield a thin-section final product. This involves multiple working processes and can, therefore, be very costly as well as inefficient in terms of energy consumption. However, the production of thin layers and mesh products is by far the biggest industrial use of electroforming [64,65]. Different stent strut thicknesses have been reported in literatures from 75 to 200 μm [66]. The electroforming process has the potential of producing layers with different thicknesses ranging from 10 μm to 5 mm, therefore, the primary objective was to produce iron foils, about 100–200 μm in thickness, by a developed electroforming apparatus and to study the effect of deposition parameters on microstructure, mechanical properties and *in vitro* degradation behavior of the fabricated foils. The developed electroforming set-up was based on the use of a ferrous chloride-calcium chloride electrolyte, a Ti6al4V alloy as substrate and an Armco^®^ iron sheet as anode. The electroforming parameters were adjusted to obtain iron foils with low surface roughness. The produced foils were annealed at 550 °C for 1 h to decrease the internal stress resulted from electrodeposition. Microstructural characterization of the iron foils showed fine grains even after annealing with an average grain size of 6 μm [23]. The microstructure of annealed electroformed iron and commercial Armco^®^ iron are presented in Figure 6. As shown in the figure, electroforming produced a much finer microstructure compared to the conventional casting and thermomechanical process (6 μm *versus* 40 μm).

**Figure 7:**
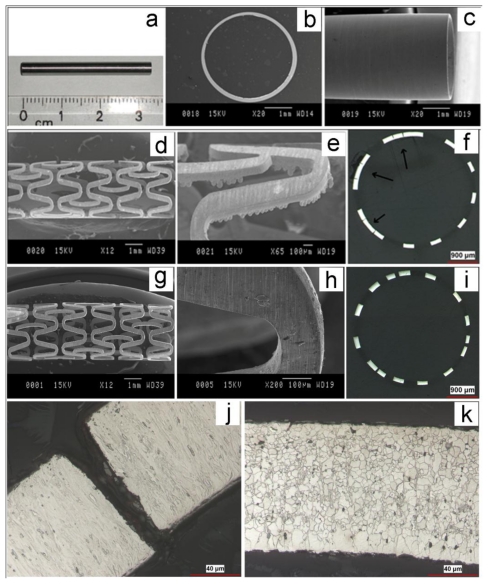
Microstructure of iron stents at different processing steps: (**a**–**c**) as ground minitube; (**d**–**f**) as laser cut minitube; (**g**–**i**) final stent; (**j**) high magnification cross section of as laser cut minitube; and (**k**) high magnification cross section of final stent.

One of the advantages of electroforming is its specific capability to produce thin walled cylinders, without a joint line [64]. This property cannot be obtained by other fabrication methods including casting and powder metallurgy. Therefore, this process could be potentially used to produce stent minitubes and even stent structures in their final form. The feasibility of developing an electroforming process to produce iron minitubes for biodegradable stents was subsequently investigated by the present authors. In this part, an electroforming process was firstly developed to produce iron minitubes for stents. Cylindrical disposable tin (Sn) substrate (Ø = 3 mm) was used as cathode on which iron tubes were electroformed. After electroforming, the minitubes were heated at 300 °C for 5 min to melt and remove the substrate. Iron tubes were then ground to improve the surface roughness and to remove the traces of Sn. Figure 7 shows the microstructure of iron stent produced from electroformed minitubes at each processing step. Figure 7a–c shows that grinding improved the surface quality of the electroformed minitube and removed the traces of Sn. The minitubes were cut into slotted-tube stents using laser cutting machine. Laser cutting parameters were the same applied for laser cutting of Apolo 316L SS stent with the same stent design. The structure of laser cut minitube is presented in Figure 7a,b. The cutting burrs could be observed in image Figure 7b. Figure 7c demonstrates the cross section of cut minitube in which the arrows show the undesired parts after cutting. As-cut tubes were then annealed at 550 °C in high purity Ar atmosphere for 1 h to relieve residual stress from electroforming and laser cutting. Acid pickling of annealed minitubes was performed in a pickling solution in ultrasonic bath to remove the undesired parts of laser cutting well as laser cutting burrs. The results (Figure 7g–i) showed that iron stents could be successfully fabricated from electroformed minitube using laser cutting method. The microstructure of iron cut minitube (Figure 7j) showed the formation of columnar grains which are typical in electrodeposited metals. The columnar grains recrystallized to fine equiaxed grains with the average size of 5 μm upon annealing as seen in Figure 8k. A finer grain size reduces the risk of a stent strut breaking and could potentially provide high mechanical properties for the electroformed iron stents. Generally, the wall thickness of stent tubes should contain a minimum of 8 to 10 grains [58]. Therefore, if the strut thickness of a stent is 100 μm, the average grain size should be 10–12.5 μm. It was shown that electroformed iron recrystallized with the average grain size of 5 μm which is well below the stent grain size specification. However, different studies [58,67,68] have shown that the grain number requirement is not always achieved for 316L SS and could sometimes lead to the failure of the stent.

## 5. Conclusive Remarks

Development of biodegradable metallic implants has revolutionized the concept of biomaterials from purely mechanical replacement devices towards true biological solutions. In this context, metallic biodegradable stents have been introduced and investigated as possible alternatives for permanent stents. Biodegradable stents provide mechanical support to the diseased artery during arterial healing and remodeling while degrading gradually and are eventually eliminated from the body. Iron and magnesium are the two available elements for such applications as they are both biocompatible and possess interesting mechanical properties for load bearing stent application. Since 2001, different iron and magnesium based biodegradable stents have been implanted in animal studies and more recently in humans in clinical investigations in order to find the suitable candidate. Although the implantation results have shown that both iron and magnesium based biodegradable stents were biocompatible compared to the currently available permanent stents, the degradation rate of these stents is yet to be modified. While the *in vivo* degradation rate of iron stent was too slow, magnesium alloy stents have shown too rapid *in vivo* degradation. Therefore, investigations are still ongoing in this field in order to modify the degradation rate of Fe and Mg-based stents through changing the alloys composition and microstructure. In fact, the objectives targeted in the development of the next generation of biodegradable metallic stents are controllable degradation rate, prolonged mechanical stability and further reduction on neointimal hyperplasia. These objectives could be mainly attained by designing new alloy compositions and microstructures, new stent strut design and use of anti-proliferative drug coatings. New advancements in materials science can be applied to combine new alloys with sophisticated stent designs resulting in minimal radial profiles and tissue contact while maintaining adequate mechanical strength and deliverability. Current research has also focused on improved stent coatings that will allow stents to serve as vehicles for local drug delivery and act as inductive scaffold for endothelial repair. The combination of micro and nanotechnology and stent design could be employed to enhance the flexibility of local drug release and demonstrate the potential to target treatment to the site of the deployed stent. The modification of the stent surface could also yield improvements in blood contacting properties, drug delivery to reduce restenosis, and accelerate endothelialization. Recently, the effect of novel metal processing techniques on the properties and degradation of biodegradable metallic materials for stent has been investigated. While the majority of stents are produced from metallic ingots fabricated by casting and thermomechanical treatments, powder metallurgy (PM) and electroforming have shown promising potential for fabrication of iron-based stent materials. Powder metallurgy Fe-Mn alloy had a faster *in vitro* degradation compared to the same alloy produced by casting because of the PM process porosity increasing the degradation rate. Electroformed iron also showed a faster *in vitro* degradation compared to Armco^®^ Fe fabricated by casting, since the electroformed material had a much finer microstructure with increased volume of grain boundaries which are more susceptible to corrosive attack. Electroforming was also used to produce iron minitubes directly on a cylindrical mandrel. The minitubes were successfully laser cut and treated to the final structure of iron stents with a fine microstructure. Thus, this new technique can be interesting to produce fine grain biodegradable iron stent minitubes with potentially interesting mechanical properties and targeted degradation in a single step fabrication process.

## Figures and Tables

**Table 1:** Mechanical properties, *in vitro* degradation rate and average grain size of different iron-based materials investigated for biodegradable stents.

Material	Yield Strength (MPa)	Tensile Strength (MPa)	Elongation (%)	*In vitro* Degradation Rate (mm y^−1^) *	Average Grain Size (μm)
316L SS: annealed (ASTM F138) [17]	190	490	40	-	12–30
Armco^®^ Fe: annealed [13,18]	150	200	40	0.19	40
Fe-35Mn alloy: annealed [19–21]	230	430	30	0.44	<100
Fe-10Mn-1Pd alloy: heat treated [22]	850–950	1450–1550	2–8	-	-
Electroformed Fe: annealed at 550 °C [23,24]	270	290	18	0.46–1.22	2–8
Fe alloyed by different elements (Mn, Co, Al, W, Sn, B, C and S): as cast [25]	100–220	190–360	12–23	0.10–0.17	100–400
Fe-30Mn-6Si alloy: solution treated [26]	180	450	16	0.30	<100
Nanocrystalline Fe: ECAP, 8 passes [27]	-	250–450	-	0.09–0.2	0.08–0.20

* The degradation rate is calculated from potentiodynamic polarization test.

**Table 2:** Mechanical properties, *in vitro* degradation rate and average grain size of different magnesium-based materials investigated for biodegradable stents.

Material	Yield Strength (MPa)	Tensile Strength (MPa)	Elongation (%)	*In vitro* Degradation Rate (mm y^−1^) *	Average Grain Size (μm)
316L SS: annealed (ASTM F138) [17]	190	490	40	-	12–30
Pure Mg: as cast [18,41]	20	86	13	407	-
WE43 alloy: extruded T5 [41]	195	280	2	1.35	10
AM60B-F: die cast [18,41,42]	-	220	6–8	8.97	25
ZW21: extruded [43–45]	200	270	17	-	4
WZ21: extruded [43–45]	140	250	20	-	7

* The degradation rate is calculated from potentiodynamic polarization test.
